# MIMIC-IV database: should serum osmolality be a critical factor in assessing prognosis for adult patients following hemorrhagic stroke surgery?

**DOI:** 10.1097/MD.0000000000046775

**Published:** 2026-01-02

**Authors:** Zhengbo Yuan, Zhanxiang Wang

**Affiliations:** a Department of Neurosurgery, Xiamen Key Laboratory of Brain Center, The First Affiliated Hospital of Xiamen University, School of Medicine, Xiamen University, Xiamen, P. R. China; b School of Medicine, Xiamen University, Xiamen, P. R. China; c Department of Neurosurgery and Department of Neuroscience, Fujian Key Laboratory of Brain Tumors Diagnosis and Precision Treatment, Xiamen Key Laboratory of Brain Center, The First Affiliated Hospital of Xiamen University, School of Medicine, Xiamen University, Xiamen, P. R. China.

**Keywords:** hemorrhagic stroke, in-hospital all-cause mortality, MIMIC-IV database, neuro intensive care unit, serum osmolality

## Abstract

Osmotherapy is commonly applied to postoperative patients with hemorrhagic stroke (HS) in the neuro-intensive care unit, necessitating precise monitoring of internal environment parameters such as serum osmolality. Although serum osmolality imbalances are associated with poor outcomes, definitive evidence regarding its prognostic significance in postoperative HS patients remains limited. This study analyzes the correlation between serum osmolality and in-hospital all-cause mortality in postoperative HS patients based on a large sample of postoperative HS patients from the Medical Information Mart for Intensive Care-IV database. A retrospective cohort of 342 HS patients from Medical Information Mart for Intensive Care-IV was analyzed. Consensus clustering was applied to group patients into 6 clusters, with COX proportional hazards regression models used to assess the relationship between serum osmolality and in-hospital mortality. Restricted cubic spline curves were drawn to evaluate the non-linear associations between variables and outcomes. Survival analysis was performed using the Kaplan–Meier method to estimate patient outcomes across groups, and intergroup differences were statistically compared with the log-rank test. Subgroup analyses were conducted to assess differences across various populations. The study included 342 patients, with consensus clustering identifying 6 groups. A significant difference in survival was found between Cluster 4 and the combined group (Cluster 1 + 2 + 3 + 5 + 6), with Cluster 4 showing a lower mortality rate (Log-rank *P* = .042). COX regression models adjusting for age, gender, comorbidities, and common clinical scores consistently showed that Cluster 4 had a lower risk of mortality. Furthermore, serum osmolality was not found to significantly influence mortality risk after adjusting for potential non-linear effects (*P* = .742). Serum osmolality does not appear to be a strong predictor of mortality in postoperative patients with surgically treated HS in the neuro-intensive care unit. Age (>65 years), gender, absence of hypertension, lack of hypertonic therapy, no invasive mechanical ventilation, and no diuretic use were significant factors influencing mortality risk.

## 1. Introduction

Hemorrhagic stroke (HS) primarily consists of spontaneous intracerebral hemorrhage (SICH) and non-traumatic subarachnoid hemorrhage (NSAH). SICH, characterized by spontaneous rupture of cerebral vessels leading to parenchymal hematoma, represents the second most prevalent stroke subtype after ischemic stroke (IS).^[1]^ It accounts for 10% to 30% of stroke admissions in Western nations, with a significantly higher prevalence in China, constituting 18.8% to 47.6% of total stroke cases.^[2]^ NSAH is a stroke subtype characterized by vascular rupture at the base or surface of the brain, causing blood to enter the subarachnoid space and induce clinical symptoms, accounting for 5% to 10% of all stroke types.^[3]^ Surgical intervention for hematoma evacuation has remained a cornerstone therapeutic approach in HS management.^[4]^ The personalized development of perioperative management strategies for HS presents an unavoidable challenge for clinicians worldwide.

Serum osmolality quantifies the solute concentration in extracellular fluid, predominantly determined by sodium, glucose, and blood urea nitrogen (BUN). This parameter evaluates both fluid-electrolyte homeostasis and renal efficiency, representing a key biomarker for systemic hydration assessment. Direct measurement of plasma osmolality is the gold standard for reflecting the hydration status of the body.^[5]^ Previous studies have shown that dehydration manifested by hyperosmolality is an important risk factor affecting the prognosis of intensive care unit (ICU) patients.^[6,7]^ However, due to the complexity of detection methods, direct measurement of plasma osmolality often imposes greater time and economic burdens on patients, rendering it less utilized in routine clinical practice. Given these limitations, current clinical practice predominantly employs calculated serum osmolality derived from sodium, potassium, glucose, and BUN levels as an indirect estimation method (Table 1).^[8,9]^ Currently, the ESPEN (European Society for Clinical Nutrition and Metabolism) formula has been recommended as a feasible method for clinically assessing dehydration.^[10,11]^ In subsequent studies, the formula has been gradually optimized to make its calculation results more reflective of actual conditions.^[12]^

**Table 1:** Formulas for serum osmolarity calculation.^[8,9]^

Formula	Units
2 × [(Na^+^) + (K^+^)] + plasma glucose + BUN	All in mmol/L
2 (Na^+^) + plasma glucose + BUN	All in mmol/L
2 (Na^+^)* + plasma glucose**/18 + BUN**/2.8	*mmol/L, **mg/dL

BUN = blood urea nitrogen, K^+^ = potassium, Na^+^ = sodium.

Currently, with the updating of medical concepts and advancements in multimodal monitoring technologies, clinicians select postoperative transitional specialty care in a Neuro ICU environment based on intraoperative conditions of SICH patients.^[13,14]^ In the Neuro ICU, postoperative patients with surgically treated HS often experience abnormalities in serum osmolality due to intracranial vascular rupture, post-surgical blood-brain barrier disruption, and the application of osmotherapy. Previous studies have shown that serum osmolality plays a crucial role in reflecting the internal environment of patients and in predicting the prognosis of HS patients.^[7,15]^ Therefore, exploring the relationship between serum osmolality and mortality in postoperative patients with surgically treated HS in the Neuro ICU setting is of significant clinical relevance. We used a large single-center sample of postoperative patients with surgically treated HS from the Medical Information Mart for Intensive Care-IV (MIMIC-IV) database for a retrospective study to investigate the relationship between serum osmolality levels and mortality risk in these patients.

## 2. Materials and methods

### 2.1. Data sources

The MIMIC-IV (version 3.1), a publicly available database sourced from the electronic health record of the Beth Israel Deaconess Medical Center. Information available includes patient measurements, orders, diagnoses, procedures, treatments, and deidentified free-text clinical notes. The MIMIC-IV is contemporary, containing information from 2008 to 2019. And MIMIC-IV incorporates new precise digital information sources such as the electronic medicine administration record. The MIMIC-IV also establishes a modular organization of the constituent data, allowing linking of the database to external departments and distinct modalities of data. MIMIC-IV is intended to support a wide array of research studies and educational material, helping to reduce barriers to conducting clinical research (https://doi.org/10.1038/s41597-022-01899-x). Our researchers have completed the online training program from the National Institutes of Health (NIH) and obtained access rights to the raw data (certification ID: 68740409) (supplementary file 1, Supplemental Digital Content, https://links.lww.com/MD/Q990). The requirement of ethical approval for this study was waived by Ethics Committee for Clinical Research at the First Affiliated Hospital of Xiamen University because the data were accessed from MIMIC-IV.

### 2.2. Study design and objects

**Figure 1.**
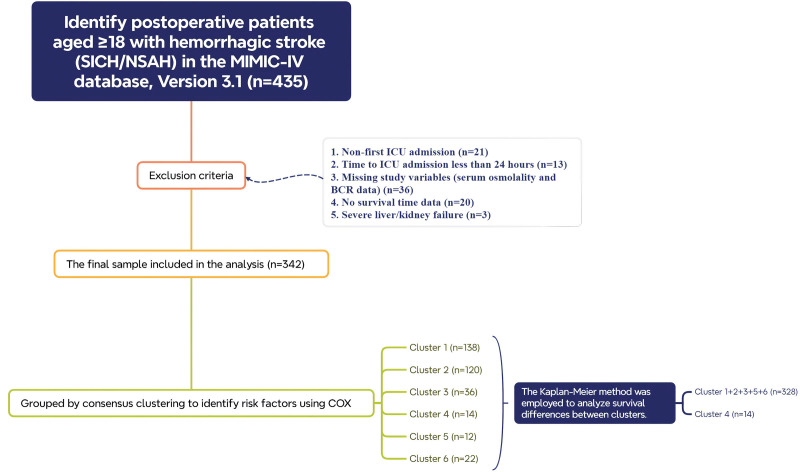
Flow chart of the design of this study. BCR = the blood urea nitrogen-to-creatinine ratio, ICU = intensive care unit, MIMIC-IV = The Medical Information Mart for Intensive Care-IV, NSAH = non-traumatic subarachnoid hemorrhage.

Collection of postoperative patients with surgically treated HS according to the International Classification of Diseases (ICD-11: 8B00*; ICD-11: 8B01*; ICD-11: 8B0Z*; ICD-11: XY7V*) (https://icd.who.int/browse/2025-01/mms/en). The flowchart of the study is shown in Figure 1. In this study, we excluded adult patients who were not first-time Neuro ICU admissions or whose Neuro ICU stay was shorter than 24 hours. We also excluded those with severe hepatic or renal failure, as well as patients lacking key study variables or survival data. The grouping in this study was performed using consensus clustering. This method involves integrating multiple clustering results to enhance the stability and accuracy of the clustering process, ensuring more reliable and reproducible groupings.

### 2.3. Formulas and outcomes

Serum osmolality formula^[12]^:


$$\begin{array}{l} \begin{array}{lll} & 1.88\times (Sodium\;ion+Potassium\;ion)+1.59\times \left(\frac{Glucose}{18}\right)\; & +1.08\times \left(\frac{Urea}{6}\right)+10.6.\; \end{array} \end{array}$$

Sodium and potassium concentrations were reported in international units (mmol/L), whereas glucose and urea, were expressed in conventional units. Because the calculation of osmolality requires molar concentrations, values given in conventional units (mg/dL) were converted to mol/L using the corresponding conversion factors (18 for glucose and 6 for urea).

The in-hospital mortality was the outcome. For participants who survived, the follow-up ended when they were discharged from the hospital. For participants who died during the hospital stay, the follow-up ended at the time of death.

### 2.4. Data collection

Navicat Premium 16.0 software was utilized to gather patient data from the database across 5 domains. (1) Baseline characteristics: age, gender, weight, and race. (2) Complications: cardiac disease, chronic pulmonary disease, rheumatic disease, renal disease, liver disease, diabetes, hypertension, respiratory failure, coronary artery disease, stroke, congestive heart failure, sepsis. (3) Common disease severity score: systemic inflammatory response syndrome (SIRS), Charles comorbidity index (CCI), acute physiology score-III (APS-III), logistic organ dysfunction system (LODS) score, and overt agitation severity scale (OASS) score. (4) Laboratory examination and vital signs: neutrophils (10^^^9/L), highest lactate (mmol/L), total bilirubin (mg/dL), troponin T (ng/mL), hemoglobin (g/dL), white blood cell count (WBC, 10^^^3/µL), platelet count (10^^^3/µL), serum creatinine (mg/dL), BUN (mg/dL), albumin (g/dL), aspartate aminotransferase (AST, U/L), alanine aminotransferase (ALT, U/L), lactate (mmol/L), sodium (mmol/L), potassium (mmol/L), chloride (mmol/L), anion gap (mmol/L), pH (arterial blood gas), and PaO_2_ (mm Hg) within 24 hours after the operation. (5) Total urine volume of patients in Neuro ICU. (6) Received treatment: diuretics, vasopressors, parenteral nutrition, hypertonic therapy, and invasive ventilation. Variables related to serum osmolality were all taken in the first examination within the first day of admission to the Neuro ICU. All variables were missing less than 10%.

### 2.5. Statistical analysis

Statistical analyses were conducted using R 4.3.3. Normally distributed continuous variables are reported as mean (standard deviation) and compared using the *t* test. Skewed continuous variables are presented as median (interquartile range, Q3) and compared using the Mann–Whitney *U* test. Categorical variables are expressed as frequencies (proportions), with differences between groups assessed using the chi-square test.

A univariate COX regression model was employed to analyze the relationship between serum osmolality and in-hospital mortality risk in postoperative patients with surgically treated HS following their first admission to the Neuro ICU. Potential covariates with *P* < .05 in the univariate COX regression analysis were identified as confounding factors requiring adjustment. Univariate and multivariate COX regression models were applied to examine the relationship between serum osmolality and hospital mortality among postoperative patients with surgically treated HS. Subgroup analyses were stratified by age, sex, hypertension, diabetes, hyperosmolar therapy, invasive mechanical ventilation, diuretic use, and parenteral nutrition. Hazard ratios (HR) and 95% confidence intervals (CI) were calculated.

In our study, we employed 5 models to control for confounding factors. Model 1: unadjusted; Model 2: adjusted for age and gender; Model 3: adjusted for age, gender, and chronic conditions; Model 4: adjusted for age, gender, chronic conditions, and clinical scores (SIRS, OASS, LODS, APS III, CCI); Model 5: adjusted for age, gender, chronic conditions, highest creatinine, anion gap, and clinical scores (SIRS, OASS, LODS, APS III, CCI). Restricted cubic spline (RCS) curves were plotted within the framework of Model 5 to further assess nonlinear associations between study variables and outcomes.

## 3. Results

### 3.1. Consensus clustering results

**Figure 2.**
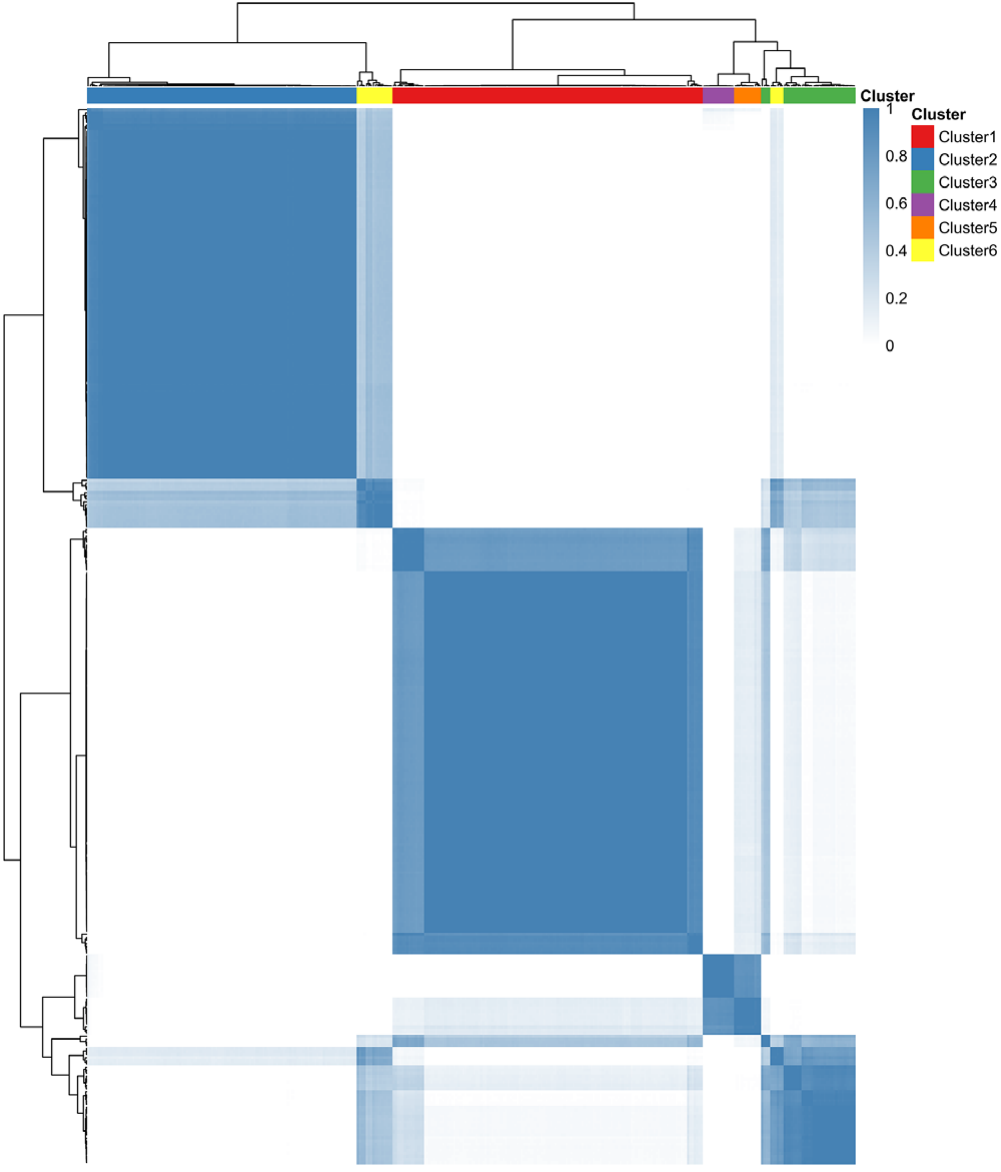
Consensus clustering of samples. Heatmap displaying the consensus matrix for 6 clusters, labeled Cluster1 through Cluster 6. The color scale on the right represents the consensus values, ranging from 0 (white) to 1 (dark blue), indicating the degree of agreement between the samples within each cluster.

**Figure 3.**
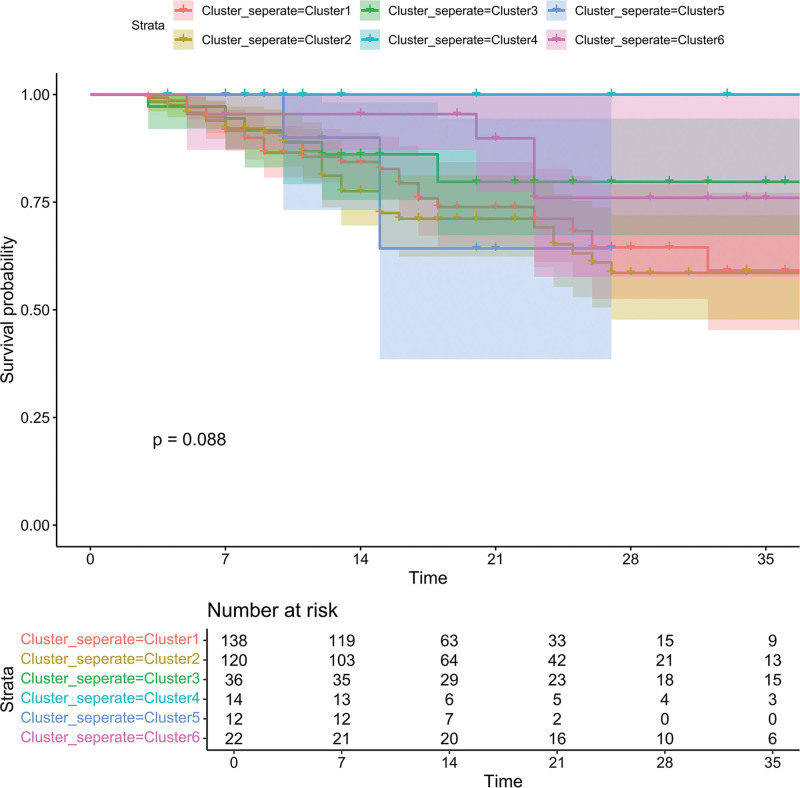
Survival curves for each cluster. Kaplan–Meier survival curves are shown for each of the 6 clusters (Cluster1 through Cluster 6), with the x-axis representing time (in days) and the y-axis indicating the survival probability. The number of patients at risk at different time points is provided for each cluster. The *P* value of the overall comparison is indicated as *P* = .088.

Using a consensus clustering method, we set the cluster number range (k = 2 to k = 8) and evaluated clustering stability through repeated subsampling. We identified k = 6 as the optimal cluster number (Fig. 2). The reliability of each k-value was assessed using consensus matrix consistency scores (range: 0–1). For k = 6, the score exceeded 0.8, indicating stable subgroup partitioning. The 6 independent subgroups (Clusters 1–6) underwent Kaplan–Meier survival analysis, with log-rank test *P* calculated (Fig. 3). Based on similarities in survival characteristics across clusters, Clusters 1, 2, 3, 5, and 6 were merged into one group, while Cluster 4 remained separate, resulting in 2 final clusters for subsequent analysis. The actual serum osmolality conditions represented by each cluster are shown in Table 2.

**Table 2:** Characteristics and outcomes of participants between cluster 4 and cluster 1 + 2 + 3 + 5 + 6.

Characteristics	Total (n = 342)	Cluster 1 + 2 + 3 + 5 + 6 (n = 328)	Cluster 4 (n = 14)	*P*
General characteristics				
Age (yr)	66.5 ± 11.5	66.8 ± 11.2	60 ± 10.0	.05
Gender				
Male	178 (52)	171 (52)	7 (50)	.81
Female	164 (48)	157 (48)	7 (50)	.81
Race				
White	245 (72)	235 (72)	10 (71)	.90
Black/African American	70 (20)	68 (21)	2 (14)	.35
Unknown	20 (6)	18 (5)	2 (14)	.07
Asian – Chinese	15 (4)	15 (5)	0 (0)	.11
Hispanic/Latino – Puerto Rican	10 (3)	8 (2)	2 (14)	**.01** *
Serum osmolality and urine volume				
Serum osmolality (mmol/L)	316.82 ± 20.19	-	312.81 ± 18.32	.52
Total Urine Output (mL)	23135 ± 15000	22900 ± 14900	13395 ± 13000	**.03** *
Complications				
Has cardiac disease	106 (31)	105 (32)	1 (7)	**.01** *
Has chronic pulmonary disease	144 (42)	140 (43)	4 (29)	.29
Has rheumatic disease	17 (5)	17 (5)	0 (0)	.23
Has renal disease	45 (13)	45 (14)	0 (0)	.08
Has liver disease	61 (18)	57 (17)	4 (29)	.14
Has diabetes	89 (26)	86 (26)	3 (21)	.65
Has hypertension	185 (54)	178 (54)	7 (50)	.70
Has respiratory failure	120 (35)	116 (35)	4 (29)	.63
Has coronary artery disease	68 (20)	67 (20)	1 (7)	.16
Has stroke	1 (0)	1 (0)	0 (0)	.99
Has congestive heart failure	51 (15)	51 (16)	0 (0)	.10
Has sepsis	61 (18)	60 (18)	1 (7)	.12
Laboratory examination and vital signs				
Neutrophils (10^9^/L)	15.23 ± 9.1	15.0 ± 8.9	9.58 ± 6.37	**.04** *
Highest lactate (mmol/L)	4.21 ± 3.51	4.1 ± 3.4	3.02 ± 1.97	.12
Total bilirubin (mg/dL)	3.24 ± 6.49	3.2 ± 6.4	0.64 ± 0.6	**.03** *
Hemoglobin (g/dL)	12.4 ± 2.1	12.2 ± 2.0	10.7 ± 2.1	.08
WBC (10^3^/µL)	11.2 ± 6.3	11.0 ± 6.2	9.9 ± 4.3	.29
Platelet Count (10^3^/µL)	210 ± 75	207 ± 74	176 ± 70	.15
Serum Creatinine (mg/dL)	1.92 ± 1.93	1.90 ± 1.90	0.97 ± 0.35	**.01** *
BUN (mg/dL)	40.9 ± 30.5	40.5 ± 30.0	20.8 ± 8.6	**.02** *
Albumin (g/dL)	3.39 ± 0.68	3.37 ± 0.67	3.46 ± 0.64	.79
AST (U/L)	276.51 ± 771.62	273 ± 760	168.55 ± 295.84	.28
ALT (U/L)	88.4 ± 105.2	87 ± 104	49.2 ± 56.1	.24
Lactate (mmol/L)	4.21 ± 3.51	4.1 ± 3.4	3.02 ± 1.97	.18
Sodium (mmol/L)	138.2 ± 5.7	138 ± 5.6	136.9 ± 5.2	.43
Potassium (mmol/L)	4.2 ± 0.8	4.1 ± 0.8	4.0 ± 0.6	.76
Chloride (mmol/L)	103.6 ± 5.1	103.4 ± 5.0	104.1 ± 5.0	.82
Anion gap (mmol/L)	18.76 ± 4.79	18.7 ± 4.7	18.64 ± 3.34	.96
pH (arterial blood gas)	7.49 ± 0.07	7.49 ± 0.07	7.48 ± 0.07	.83
PaO_2_ (mm Hg)	318.48 ± 129.38	316 ± 128	309.79 ± 106.43	.75
Received treatment				
Diuretics				
Yes	50 (15)	48 (15)	2 (14)	.94
No	292 (85)	280 (85)	12 (86)	.94
Vasopressors				
Yes	30 (9)	28 (9)	2 (14)	.37
No	312 (91)	300 (91)	12 (86)	.37
Parenteral nutrition				
Yes	40 (12)	37 (11)	3 (21)	.11
No	302 (88)	291 (89)	11 (79)	.11
Hypertonic therapy				
Yes	20 (6)	19 (6)	1 (7)	.81
No	322 (94)	309 (94)	13 (93)	.81
Invasive ventilation				
Yes	10 (3)	9 (3)	1 (7)	.30
No	332 (97)	319 (97)	13 (93)	.30
Common disease severity score				
LODS score	6.96 ± 3.76	6.95 ± 3.75	6.38 ± 3.57	.78
OASS score	38.01 ± 9.01	38.00 ± 9.00	37.23 ± 9.81	.84
CCI score	5.65 ± 3.11	5.64 ± 3.10	3.00 ± 3.01	.09
APS III score	63.61 ± 29.16	63.50 ± 29.00	62.00 ± 25.82	.82

Values are presented as mean ± standard deviation or n (%).

ALT = alanine aminotransferase, APS-III = acute physiology score-III, AST = aspartate aminotransferase, BUN = blood urea nitrogen, CCI = Charles comorbidity index, LODS = logistic organ dysfunction system, OASS = overt agitation severity scale, WBC = white blood cell count.

*P* with * in bold indicate statistical significance (*P* < .05).

### 3.2. Baseline data analysis

In our study, a total of 342 patients participated. Consensus clustering was used to divide the patients into 6 clusters. The median age of these patients was 66.5 years, with 52% being male, and the majority of patients were White (71.6%). The baseline characteristics of the patients in each group are shown in Table 2.

### 3.3. Prognostic analysis

**Figure 4.**
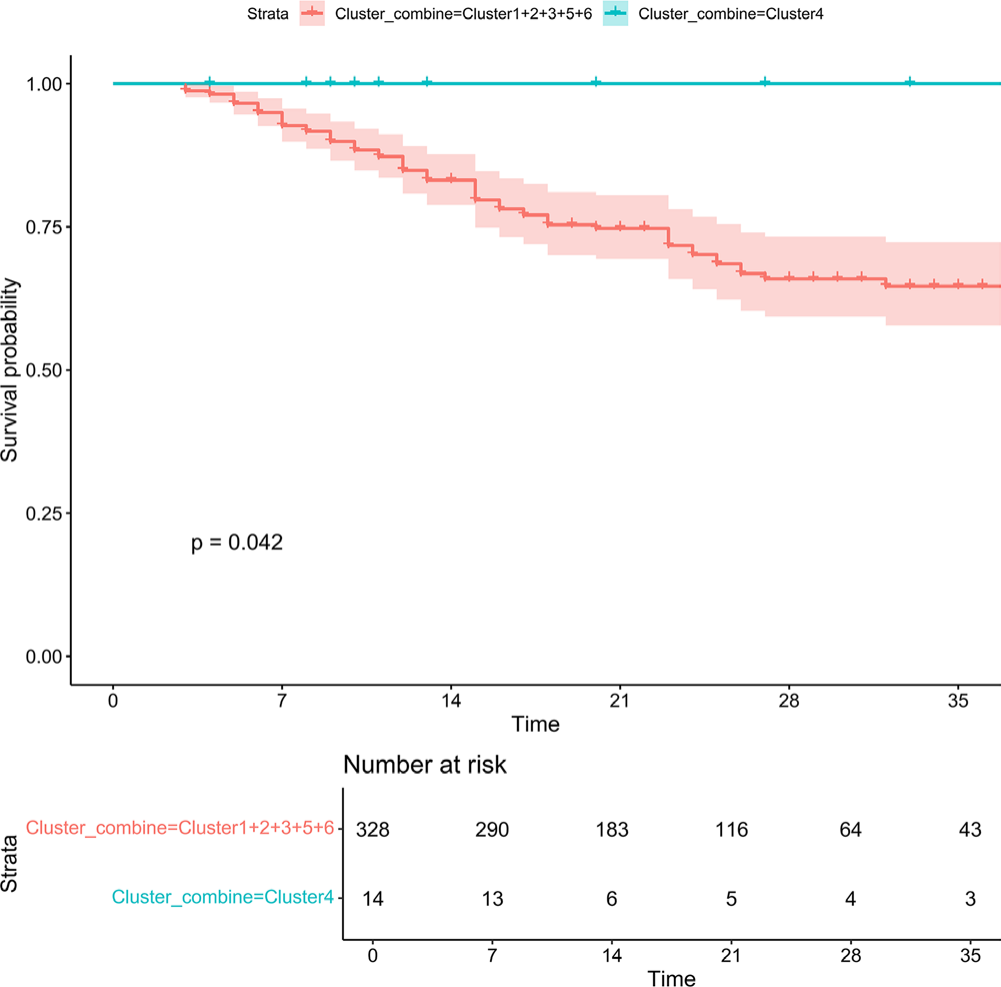
Survival comparison between Cluster 4 and the combined clusters (Cluster 1 + 2 + 3 + 5 + 6). Kaplan–Meier survival curves for the combined clusters (Cluster 1 + 2 + 3 + 5 + 6) and Cluster 4 are shown. The x-axis represents time (in days), and the y-axis indicates the survival probability. The number of patients at risk at different time points is provided for each group. The *P* value of the comparison is *P* = .042.

Descriptive statistical analysis of the prognostic information for each group of patients was performed. Among all clusters, the median length of stay in the Neuro ICU was 20.15 days, and the in-hospital mortality rate was 0.2%. The survival difference between the combined group (Cluster 1 + 2 + 3 + 5 + 6) and Cluster 4 showed that Cluster 4 had a significantly higher survival difference compared to the combined group (log-rank *P* = .042) (Fig. 4; Table 3). The differences between Cluster 4 and Cluster 1 + 2 + 3 + 5 + 6 were compared, and variables to be included in the model were determined based on actual clinical significance.

**Table 3:** Outcomes of cluster of patients.

Characteristics	Total (n = 342)	Cluster 1 + 2 + 3 + 5 + 6 (n = 328)	Cluster 4 (n = 14)	*P*
Length of stay (d)	20.15 ± 18.09	19.7 ± 17.5	37.29 ± 53.48	**.02** *
In-hospital mortality	0.2 (1)	0.19 (1)	0.08 (0)	**.43** *
Mortality 7-day	0.06 (0)	0.05 (0)	0 (0)	1.00
Mortality 90-day	0.2 (1)	0.19 (1)	0.08 (0)	**.43** *
Mortality 1-year	0.2 (1)	0.19 (1)	0.08 (0)	**.43** *

Values are presented as mean ± standard deviation.

*P* with * in bold indicate statistical significance (*P *< .05).

### 3.4. Correlation analysis

**Figure 5.**
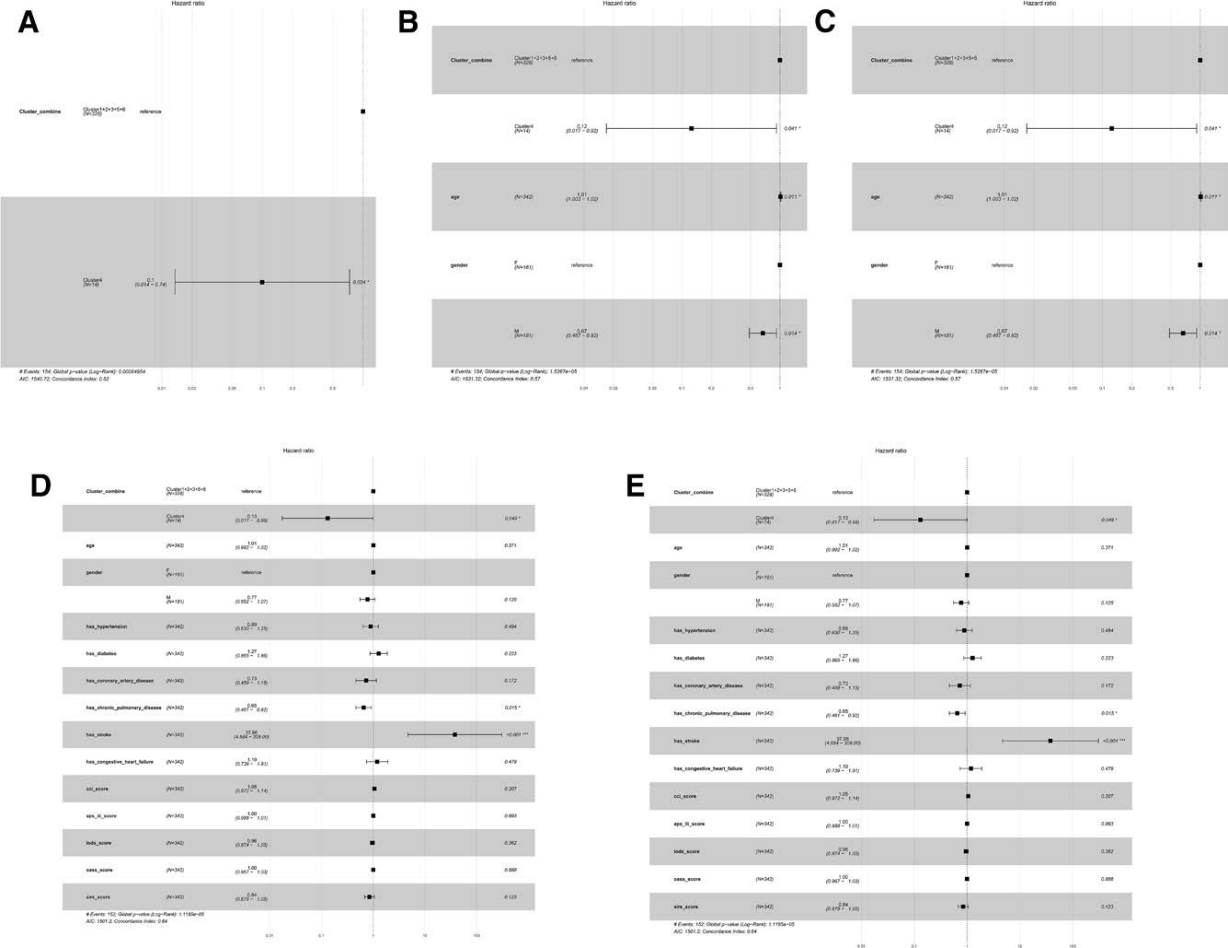
Model calibration process. A: Model 1 (unadjusted). B: Model 2 (adjusted for age and gender). C: Model 3 (further adjusted for clinical comorbidities). D: Model 4 (incorporated common clinical scoring systems). E: Model 5 (fully adjusted model).

Using Cluster 4 (the subgroup with the lowest in-hospital all-cause mortality) as the reference group, a COX proportional hazards regression model was constructed to analyze the association between serum osmolality and mortality in postoperative patients with surgically treated HS. Model 1 (unadjusted) (Fig. 5A): Only the grouping variable was included in the COX analysis. The results showed that Cluster 4 had a significantly lower event risk compared to the combined group, with HR = 0.10 (95% CI: 0.014–0.74, *P* = .024). The model’s C-index was 0.52, and the AIC value was 1540.72, indicating that both the model’s discriminative power and fit were limited. Model 2 (adjusted for age and sex) (Fig. 5B): Age and sex were added to the baseline Model 1. After controlling for these 2 common confounders, Cluster 4 retained a significant protective effect (HR = 0.12, 95% CI: 0.017–0.92, *P* = .041). Additionally, age exhibited a positive association in this model (HR = 1.01, *P* = .011), indicating higher risk with advancing age. Gender (male vs female) also had some influence (HR = 0.67, *P* = .014). After correction, the model’s discriminative power improved (C-index = 0.57), the AIC decreased to 1531.32, and the fit also improved. Model 3 (comorbidity-adjusted) (Fig. 5C): This model expanded adjustments to include age, sex, and major comorbidities (stroke, chronic pulmonary disease, diabetes, hypertension, and coronary artery disease). The C-index increased to 0.63, and AIC further decreased to 1526.28 in this model. The results show that some chronic diseases, especially stroke, have a significant impact on risk (HR > 30); the relative risk for Cluster 4 remains significantly reduced (HR = 0.11, *P* = .032), indicating that the grouping still has independent predictive value for prognosis.

Model 4 (inclusion of common clinical scores) (Fig. 5D): Based on the previous models, severity or comorbidity scores such as SIRS, OASS, LODS, APS III, and CCI were added. The C-index increased to 0.64, and the AIC decreased to 1501.20, the lowest among all models, indicating that this model has the best fit and higher discriminative ability. At this point, the HR for Cluster 4 remained at 0.13 (*P* = .049), suggesting that after controlling for more clinical scores, Cluster 4 still significantly outperforms the combined group. Model 5 (Fully adjusted model) (Fig. 5E): Finally, key laboratory indicators such as highest creatinine and anion gap were included. The results showed that this model’s C-index was 0.63, and the AIC was 1504.56, similar to Model 4; the protective effect of Cluster 4 remained significant (HR = 0.13, *P* = .048). Additionally, similar to comorbidities and clinical scores, risk factors like stroke still occupied very high risk ratios (Table 4).

**Table 4:** Cox proportional hazard ratios (HR) for different clusters combination comparison during follow-up.

	Model 1		Model 2		Model 3		Model 4		Model 5	
	HR (95% CI)	*P*	HR (95% CI)	*P*	HR (95% CI)	*P*	HR (95% CI)	*P*	HR (95% CI)	*P*
28 days death										
Cluster 1 + 2 + 3 + 5 + 6	[Reference]		[Reference]		[Reference]		[Reference]		[Reference]	
Cluster 4	0.10 (0.014–0.74)	0.024	0.12 (0.017–0.92)	0.041	0.11 (0.015–0.83)	0.032	0.13 (0.017–0.99)	0.049	0.13 (0.017–0.98)	0.048

*P* denotes the significance level (*P* < .05, *P* < .01, *P* < .001); *P* for trend represents the global *P* (Log-Rank) derived from the figures. Model adjustments include: Model 1 – unadjusted; Model 2 – adjusted for age and gender; Model 3 – adjusted for age, gender, and chronic conditions; Model 4 – adjusted for age, gender, chronic conditions and clinical scores (SIRS, OASS, LODS, APS III, CCI); Model 5 – adjusted for age, gender, chronic conditions, highest creatinine, anion gap, and clinical scores (SIRS, OASS, LODS, APS III, CCI).

ALT = alanine aminotransferase, APS-III = acute physiology score-III, AST = aspartate aminotransferase, BUN = blood urea nitrogen, CCI = Charles comorbidity index, CI = confidence interval, HR = hazard ratio, LODS = logistic organ dysfunction system, OASS = overt agitation severity scale, WBC = white blood cell count.

**Figure 6.**
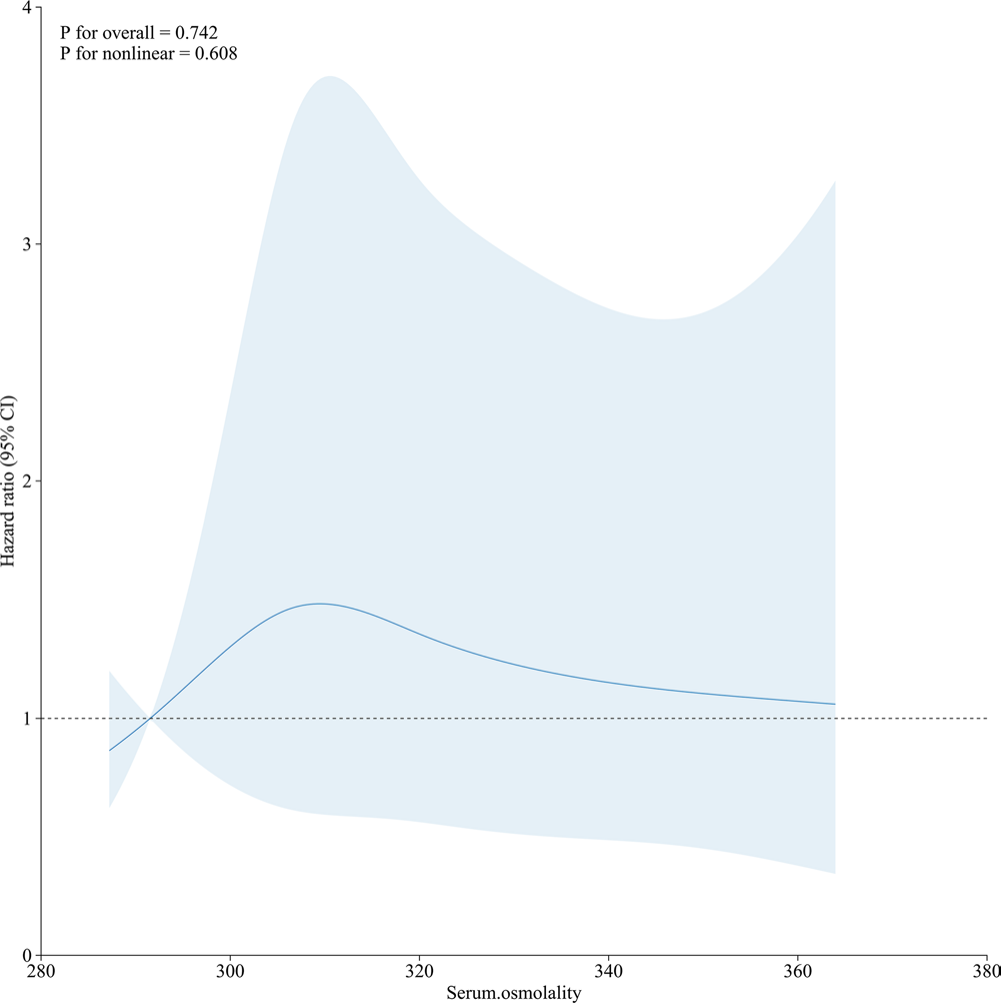
The correlation between serum osmolality and the HR of in-hospital all-cause mortality in HS postoperative patients. HR = hazard ratio, HS = hemorrhagic stroke.

Within the framework of COX proportional hazards Model 5, we plotted RCS curves to visualize the nonlinear relationship between continuous serum osmolality and mortality risk (Fig. 6). The curve results showed *P* for overall = .742, indicating that when considering all possible nonlinear effects in the model, the effect of serum osmolality on the hazard ratio was not statistically significant. *P* for nonlinear = .608, which suggests there is no significant evidence of a nonlinear association between serum osmolality and hazard rate. Therefore, serum osmolality does not have a significant nonlinear effect on risk events. Given the nonsignificant *P* values for both overall and nonlinear effects, serum osmolality did not emerge as a robust predictor in this model.

### 3.5. Subgroup analysis

Stratified subgroup analyses were performed using age, sex, hypertension, diabetes, hyperosmolar therapy, invasive mechanical ventilation, diuretics, and parenteral nutrition as stratification factors. The results revealed statistically significant differences in subgroups stratified by age (>65 years, *P* = .01), sex (*P* = .04), hypertension (*P* = .03), diabetes (*P* = .03), hyperosmolar therapy (*P* = .02), invasive mechanical ventilation (*P* = .02), and diuretics (*P* = .02) (Table 5).

**Table 5:** Subgroup analysis of the relationship between clusters and mortality by odds ratio.

Characteristics	Cluster1 + 2 + 3 + 5 + 6 (n = 328) HR (95% CI)	Cluster4 (n = 14) HR (95% CI)	*P*
Gender			
Female	0.96 (0.93, 1)	Reference	**.04** *
Male	0.94 (0.9, 0.97)	Reference	
Has hypertension			
No	0.88 (0.82, 0.94)	Reference	**.03** *
Yes	0.98 (0.95, 1.01)	Reference	
Has diabetes			
No	0.95 (0.92, 0.98)	Reference	**.03** *
Yes	0.95 (0.9, 1)	Reference	
Age > 65			
No	0.94 (0.9, 0.99)	Reference	**.01** *
Yes	0.96 (0.92, 0.99)	Reference	
Hypertonic therapy			
No	0.95 (0.93, 0.98)	Reference	**.02** *
Yes	0.84 (0.66, 1.05)	Reference	
Invasive ventilation			
No	0.95 (0.92, 0.97)	Reference	**.02** *
Yes	0.98 (0.91, 1.06)	Reference	
Diuretics			
No	0.95 (0.92, 0.98)	Reference	**.02** *
Yes	0.97 (0.86, 1.09)	Reference	
Parenteral nutrition			
No	0.97 (0.88, 1.08)	Reference	.07
Yes	0.92 (0.89, 0.95)	Reference	

CI = confidence interval, HR = hazard ratio.

*P* with * in bold indicate statistical significance (*P* < .05).

## 4. Discussion

In our study, we retrospectively analyzed the association between serum osmolality and in-hospital all-cause mortality in 342 critically ill patients after HS surgery. By progressively refining the statistical models – from simple clustering (Model 1) to incorporating age, gender, and chronic diseases (Model 2 and 3), clinical severity scores (Model 4), and additional laboratory indicators (Model 5) – we consistently found Cluster 4 significantly correlated with better survival outcomes across all analyses. As the models advanced from Model 1 through Models 2 and 3, the C-index continuously improved while the AIC decreased, indicating enhanced explanatory power regarding risk differences upon inclusion of demographic and underlying health factors. Model 4, which further incorporated clinical severity scores, showed the optimal overall performance with the highest C-index and the lowest AIC value. Adding additional laboratory indicators in Model 5 maintained the strong performance of the model without diminishing the significance of Cluster 4. Moreover, restricted cubic spline (RCS) analysis of serum osmolality in the fully adjusted Model 5 revealed no significant nonlinear relationship with postoperative mortality risk among HS surgery patients. Additionally, subgroup analysis demonstrated that advanced age (>65 years), gender, absence of hypertension, absence of hypertonic treatment, absence of invasive mechanical ventilation, and no use of diuretics were significant determinants of increased mortality risk. In contrast, the presence of diabetes, use of parenteral nutrition, and administration of hypertonic treatment showed no significant association with mortality risk.

In the Neuro ICU, osmotic therapy is regarded as an effective measure that should be promptly implemented for patients after HS surgery, irrespective of the underlying cause of increased intracranial pressure.^[16]^ Osmotic therapy can temporarily decrease intracranial volume, thus creating a critical time window for edema resolution. The primary objective of osmotic therapy is to maintain or restore sufficient cerebral blood flow, thereby alleviating the impact of postoperative cerebral edema.^[17]^ Therefore, the use of mannitol and hypertonic saline (HTS) is common practice in the Neuro ICU. Mannitol, a freely filtered, non-metabolized sugar alcohol, reduces water and sodium reabsorption in renal tubules, thereby exerting a diuretic effect. It has long been regarded as the cornerstone of treatment for cerebral edema.^[17]^ Compared to mannitol, HTS has a weaker diuretic effect, but unlike mannitol, HTS can expand the vascular content and increase blood pressure, theoretically providing a way to sustainably increase cerebral blood flow.^[18]^ The use of HTS is suggested over other therapies (e.g., mannitol) in certain neurological pathologies, as outlined in the Neurocritical Care Society’s cerebral edema guidelines, published in 2020.^[19]^

Previous studies have demonstrated that serum hyperosmolarity – particularly hypertonicity – has significant clinical implications, including neurological sequelae and increased mortality.^[11]^ Similarly, in patients with aneurysmal NSAH, hyperosmotic states are often strongly associated with higher mortality rates.^[20]^ Serum osmotic imbalance is a common finding among ICU patients and is closely associated with poor clinical outcomes.^[7,21]^ In clinical practice, the BCR is commonly used to assess internal homeostasis. However, relying solely on BCR to evaluate hydration status lacks specificity, as elevated BCR levels may also occur in patients with gastrointestinal bleeding, sepsis, major surgery, or starvation.^[22,23]^ Additionally, high-dose glucocorticoid therapy can significantly influence BCR levels.^[24]^ Chen’s research team further reported a weak correlation between BCR and increased in-hospital mortality in IS patients in the ICU, particularly among males, those with respiratory failure, malignancies, no history of liver disease, or those receiving anticoagulant therapy.^[25]^ These findings collectively underscore the limitations of using BCR alone to predict poor outcomes in stroke patients, while serum osmolality appears to offer greater prognostic value, particularly in patients with SICH.^[15]^

Therefore, it is essential to investigate the relationship between serum osmolality and mortality in postoperative patients with surgically treated HS. In our study, we observed that this association was limited in the Neuro ICU setting, where osmotic therapy is routinely administered. Under such conditions, serum osmolality may no longer serve as a strong prognostic marker for postoperative outcomes. However, this does not negate the value of monitoring serum osmolality; rather, its prognostic utility in this patient population appears to be constrained. Regardless of the hyperosmolar agent used for osmotherapy, we recommend close monitoring of fluid balance,^[26]^ as mannitol may induce significant diuresis and hypovolemia, while HTS increases intravascular volume and may result in fluid overload. Consequently, serial measurements of serum sodium and plasma osmolality are commonly performed following administration of either agent. Notably, the optimal target ranges for serum sodium and osmolality remain a subject of clinical debate.^[17,27]^

Leveraging the advantages of the MIMIC-IV database, an increasing number of clinicians are conducting data-driven analyses. Chen’s team investigated the role of serum osmolality in patients with traumatic brain injury (TBI) and examined its association with in-hospital all-cause mortality. Their findings revealed a U-shaped relationship between serum osmolality and in-hospital mortality in TBI patients, with the lowest mortality observed when serum osmolality was maintained at approximately 295.4 mmol/L.^[28]^ We hold reservations about the conclusion that serum osmolality exhibits a U-shaped relationship with in-hospital mortality in TBI patients. In the study, key confounding factors – such as total fluid intake, urine output (or other fluid losses), diuretic administration, and vasopressor therapy – showed significant differences across groups and are known to directly influence serum osmolality. However, the authors did not adequately address the challenge of standardizing these variables under clinically relevant conditions, thereby compromising the internal validity of their findings. Moreover, the RCS analysis did not report *P* values for either the overall association or nonlinearity, preventing confirmation of the statistical significance of the proposed U-shaped relationship. Finally, substantial heterogeneity in treatment protocols among TBI patients further limits the generalizability of the results. Additionally, Hu et al^[15]^ investigated the relationship linking serum osmotic parameters to mortality risk during hospitalization among patients with SICH, demonstrating that hyperosmolar derangements correlated with heightened in-hospital fatality rates in acute intracerebral hemorrhage cohorts. This has provided us with significant inspiration and has laid a solid foundation for our analysis. We acknowledge that postoperative monitoring and management of critically ill neurosurgical patients in the Neuro ICU are of paramount importance. However, previous study findings differ substantially from our current results. We attribute this discrepancy to the disruption of the blood–brain barrier and the widespread use of osmotherapy in postoperative HS patients, both of which continuously alter serum osmolality, rendering it an unreliable indicator of the patient’s intrinsic physiological status. Therefore, from a clinical perspective, serum osmolality may not serve as a dependable predictor of mortality risk in patients following HS surgery.

Our study has several limitations. First, it is a retrospective analysis based on the MIMIC-IV database, which represents data from a single center and may introduce selection bias, particularly within the Neuro ICU population. Second, important variables that could influence outcomes – such as the exact location and volume of intracranial hemorrhage – were not available in the MIMIC-IV database and were therefore excluded from our analysis. Looking ahead, identifying novel predictive factors remains essential. A deeper understanding of the relationship between dehydration and in-hospital mortality in patients with hemorrhagic stroke will require elucidation of the underlying biological mechanisms, which may necessitate the use of animal models and prospective cohort studies.

## 5. Conclusion

This retrospective study, utilizing data from postoperative patients with surgically treated HS admitted to the Neuro ICU in the MIMIC-IV database, assessed the relationship between serum osmolality and in-hospital all-cause mortality. The findings indicate that serum osmolality is not a strong predictor of mortality in this patient population. Although monitoring serum osmolality remains clinically relevant, it may not play a critical role in outcome prediction for postoperative HS patients. In contrast, factors such as advanced age (>65 years), male gender, absence of hypertension, lack of hypertonic therapy, absence of invasive mechanical ventilation, and no use of diuretics were significantly associated with increased mortality risk.

## Acknowledgments

We appreciate all the developers and researchers who contributed to the creation and maintenance of the MIMIC-IV database.

## Author contributions

**Conceptualization:** Zhanxiang Wang.

**Data curation:** Zhengbo Yuan.

**Formal analysis:** Zhengbo Yuan.

**Investigation:** Zhengbo Yuan.

**Methodology:** Zhengbo Yuan.

**Supervision:** Zhanxiang Wang.

**Validation:** Zhengbo Yuan.

**Writing – original draft:** Zhengbo Yuan.

**Writing – review & editing:** Zhanxiang Wang.

## Supplementary Material


