# Supplementary material for: MIMIC-IV database: should serum osmolality be a critical factor in assessing prognosis for adult patients following hemorrhagic stroke surgery?
Source: Medicine (Baltimore). 2026 Jan 2;105(1):e46775. doi: 10.1097/MD.0000000000046775 (PMC12778118; doi:10.1097/MD.0000000000046775)
Supplement: Supplementary file 1 [file medi-105-e46775-s001.pdf]

CITI PROGRAM

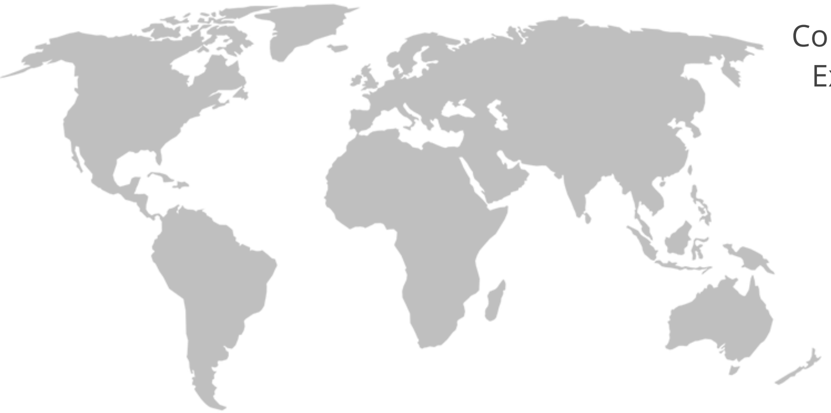

Completion Date 27-Mar-2025
Expiration Date 27-Mar-2028
Record ID 68740409

This is to certify that:

Zhengbo Yuan

Not valid for renewal of certification through CME.

Has completed the following CITI Program course:

Human Research
(Curriculum Group)
Data or Specimens Only Research
(Course Learner Group)
1 - Basic Course
(Stage)

Under requirements set by:

Massachusetts Institute of Technology Affiliates

CITI
Collaborative Institutional Training Initiative

101 NE 3rd Avenue, Suite 320
Fort Lauderdale, FL 33301 US
www.citiprogram.org

Generated on 27-Mar-2025. Verify at www.citiprogram.org/verify/?wd43ba564-9819-45c0-82c4-cdbe23f690d0-68740409
